# Aortic regurgitation following transcatheter closure of perimembranous ventricular septal defect in children: risk factors and long-term outcomes

**DOI:** 10.1007/s12928-025-01221-7

**Published:** 2025-11-26

**Authors:** Jing Liu, Nan Zhang, Jing Zhang, Bo Han, Diandong Jiang

**Affiliations:** 1 Department of Neonatology, Shandong Provincial Hospital Affiliated to Shandong First Medical University, Jinan, 250021 Shandong China; 2 Department of Cardiac Ultrasound, Shandong Provincial Hospital Affiliated to Shandong First Medical University, Jinan, 250021 Shandong China; 3 Department of Pediatric Respiratory, Shandong Provincial Hospital Affiliated to Shandong First Medical University, Jinan, 250021 China; 4 Department of Pediatric Cardiology, Shandong Provincial Hospital Affiliated to Shandong First Medical University, No.324 Jingwu Road, Jinan, 250021 China

**Keywords:** Aortic regurgitation, Ventricular septal defect, Transcatheter closure, Risk factors, Long-term outcomes

## Abstract

Postprocedural aortic regurgitation (AR) is a challenging complication following transcatheter device closure of perimembranous ventricular septal defects (pmVSD) in pediatric patients. This study aimed to identify risk factors for AR and evaluate its long-term outcomes. A total of 1,427 children who underwent successful pmVSD closure between June 2002 and August 2023 were retrospectively analyzed, with a median follow-up of 80 months (range, 12 to 248). Patients were further divided into early (2002–2016) and late (2017–2023) eras to assess temporal effects. Postprocedural AR occurred in 157 patients (11.0%), including 151 new-onset and 6 aggravated cases. AR was mostly trivial or mild, with 3 moderate cases (one requiring early valve repair). AR developed in 105 patients early after closure and in 52 during follow-up. Multivariate analysis identified repeated track creations (odds ratio [OR]: 2.000; 95% confidence interval [CI]: 1.286 to 3.112; *P* = 0.002) and opening the left disk above the aortic valve (OR: 5.071; 95% CI: 2.476 to 10.389; *P* < 0.001) as independent risk factors. Subgroup analysis showed consistent findings in the early era, whereas a smaller subaortic rim emerged as an additional risk factor in the late era. At the last follow-up, 2 patients had moderate AR, 10 mild, and 62 trivial. In conclusion, most AR cases after pmVSD closure are mild or trivial with favorable long-term outcomes. Minimizing repeated track creations and avoiding opening the left disk above the aortic valve may help reduce AR risk, whereas a smaller subaortic rim may increase its likelihood.

**Graphical Abstract:**

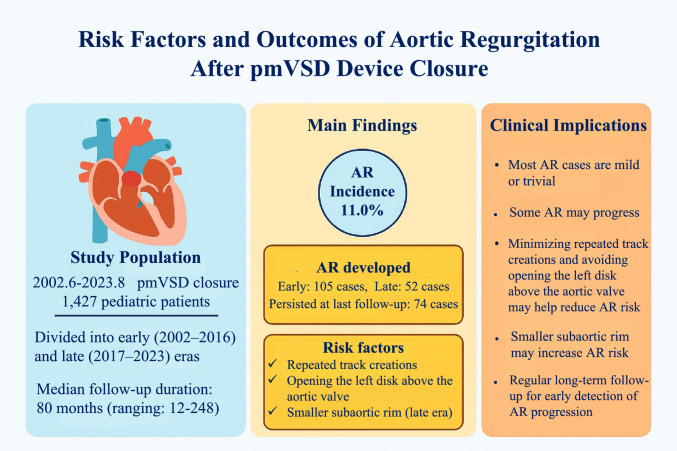

**Supplementary Information:**

The online version contains supplementary material available at 10.1007/s12928-025-01221-7.

## Introduction

Transcatheter device closure of perimembranous ventricular septal defects (pmVSD) has proven highly effective in pediatric patients [1–4]. With ongoing advancements in devices and techniques, it has become a key treatment option for children with pmVSD. [5–7] Aortic regurgitation (AR) is a noteworthy complication after closure, drawing early attention due to the aortic valve’s critical role in cardiac function. Given the proximity of pmVSDs—especially to the right coronary cusp—earlier studies focused mainly on device-related causes of postprocedural AR, leading to the development of the first clinically used Amplatzer pmVSD occluder with an eccentric design. [8]

In recent years, expanding indications have allowed closure of pmVSDs located less than 2 mm from the aortic valve, or even with right coronary cusp prolapse, which were once considered contraindications. [9, 10] Consequently, the incidence of postprocedural AR has increased. While most cases are mild, recent follow-up studies have shown that AR can progressively worsen in some patients, eventually requiring surgical intervention. [1, 2, 11] Thus, AR remains a challenging complication following VSD closure.

Currently, the reported incidence of AR following pmVSD closure varies, and the associated risk factors and outcomes remain unclear. In particular, there is a lack of large-scale studies focused on AR risk and prognosis in pediatric patients. This study aims to identify the risk factors for AR and assess its long-term outcomes in children undergoing transcatheter pmVSD closure.

## Methods

### Study design

#### Study population

This study included a cohort of consecutive pediatric patients who underwent successful transcatheter closure of pmVSD at Shandong Provincial Hospital Affiliated to Shandong First Medical University between June 2002 and August 2023. All patients had a follow-up of at least one year. The inclusion criteria for closure were: age ≥ 2 years or weight ≥ 10 kg, presence of left-to-right shunting at the ventricular level, and clinical symptoms or signs of left heart overload. Exclusion criteria: doubly committed subarterial VSD, AR greater than mild, concomitant obstructive pulmonary hypertension.

Written informed consent was obtained from all patients’ guardians, and the study protocol was approved by the institutional review board. The study adhered to the ethical principles outlined in the Declaration of Helsinki.

#### VSD closure procedure

The procedure was performed under general or local anesthesia. Left ventricular and ascending aortic angiography were used to assess the size, location, and morphology of the defect, guiding occluder selection. Devices used included symmetric, eccentric, thin-waist double-disk VSD occluders (Shanghai Shape Memory Alloy, Shanghai, China; Lifetech Scientific, Shenzhen, China; Starway Medical, Beijing, China), and the Amplatzer Duct Occluder II (ADO II) device (St. Jude Medical, St. Paul, MN). In symmetric occluders, both disks are equal in size, each 4 mm larger than the waist. Eccentric occluders have a left disk with an aortic flange of 0 or 0.5 mm and an opposite flange 5.5 or 6 mm larger than the waist. The thin-waist occluder features a left disk 8 mm larger than the waist. The right disk of both eccentric and thin-waist types is the same as that of the symmetric occluder.

Occluders were delivered via either an antegrade femoral venous approach (with an arteriovenous loop) or a retrograde femoral arterial approach. For symmetric, eccentric, and thin-waist occluders, the preferred technique was antegrade with the left disk opened on the left ventricle (LV) side of the septum; the system was then gently retracted to seat the waist within the defect, and the right disk was released in the right ventricle (RV). When safe advancement of the delivery sheath into the LV was not feasible, the left disk was temporarily unsheathed above the aortic valve to facilitate manipulation, after which the assembly was positioned so that the waist engaged the defect and the right disk was released in the RV. ADO II devices were deployed antegradely as described above (with the left disk initially opened above the aortic valve and then seated across the septum) or via a retrograde femoral arterial route. For the retrograde approach, the sheath was advanced across the aortic valve into the LV and through the defect into the RV; the right disk was opened in the RV, the waist was drawn into the defect, and the left disk was released on the LV side of the septum. Post-procedure, all patients were prescribed aspirin (3–5 mg/kg/day) for six months.

The symmetric occluder is generally preferred. If the distance from the defect to the aortic valve is less than 2 mm, an eccentric occluder is typically chosen. However, if the left disk can be placed within a membranous aneurysm, a non-eccentric occluder may still be considered, even when the distance is under 2 mm. For large membranous aneurysm-type pmVSDs with multiple outlets, either a symmetric or thin-waist occluder can be considered. The use of the ADO II generally requires a minimum distance of 3mm from the aortic valve, with a maximum defect diameter of 4mm. It has unique advantages for occluding long tubular or porous membranous aneurysm-type VSDs.

In our experience, symmetric or eccentric occluders are usually sized 2–4 mm larger than the defect diameter measured by angiography. For ADO II, the device is typically 1–2 mm larger than the defect. For thin-waist occluders, the left disk diameter should be at least equal to or greater than the aneurysm’s inlet diameter.

#### Postprocedural follow-up management

All patients received routine electrocardiography (ECG), chest X-ray, and transthoracic echocardiography (TTE) before the procedure, on the first day after the procedure, and at 1, 3, 6, and 12 months, as well as during subsequent follow-up visits. Postprocedural AR was defined as either new-onset AR or the progression of pre-existing AR.

#### Echocardiographic assessment of AR

The parasternal long-axis view is commonly used to measure the left ventricular outflow (LVOT) tract, aortic annulus, and aortic sinuses. Aortic valve leaflet thickness and morphology are assessed from this view, as well as from the parasternal short-axis and apical five-chamber views. The severity of AR is typically graded using qualitative and semi-quantitative methods, with a four-grade classification: trivial (1+), mild (2+), moderate (3+), and severe (4+). [12, 13]

**Mild AR**: The regurgitant jet appears as a narrow streak, confined to the area beneath the aortic valve, or the jet width occupies 10%-25% of the LVOT area. **Moderate AR**: The jet begins narrow and gradually widens as it extends into the LVOT, without exceeding the level of the anterior mitral valve leaflet, or occupies 25%-65% of the LVOT area. **Severe AR**: The jet may fill the entire LVOT, extending to the apex of the heart, or occupies ≥ 65% of the LVOT area. If the jet is confined to the area beneath the aortic valve, typically appearing as a single-color flow during early diastole with a normal aortic valve structure, or occupies less than 10% of the LVOT area, it is classified as **trivial AR**.

#### Statistical analysis

The variables included in the analysis were gender, age, weight, inlet and outlet diameters of VSD, subaortic rim size, membranous aneurysm, the left disk placed within the aneurysmal tissue, delivery sheath, device type and diameter, creating more than one track, occluder deployment approach, and fluoroscopic time. Data were analyzed using SPSS version 25.0. Normally distributed continuous variables are presented as mean ± standard deviation, with group comparisons conducted using the independent samples t-test. Non-normally distributed continuous variables are expressed as median (Q1, Q3), with group comparisons performed using the Wilcoxon rank-sum test. Categorical variables are reported as frequencies (%), with group comparisons made using the chi-square test or Fisher’s exact test. Collinearity diagnostics were conducted on variables with a *P*-value < 0.1 from the univariate analysis, using a condition index ≥ 30 or a variance inflation factor >5 as the threshold for identifying potential collinearity. No evidence of collinearity was found among these variables. After addressing any collinearity concerns, the variables with a P-value < 0.1 from the univariate analysis were included in a multivariate logistic regression model to identify the risk factors for postoperative AR and calculate the odds ratio (OR). A *P* value of < 0.05 was considered statistically significant.

To investigate the temporal effects of device improvement and operator experience, the study cohort was divided into an early era (2002–2016) and a late era (2017–2023). Subgroup analyses were performed using the same statistical methods.

## Results

### Study population

**Fig 1:**
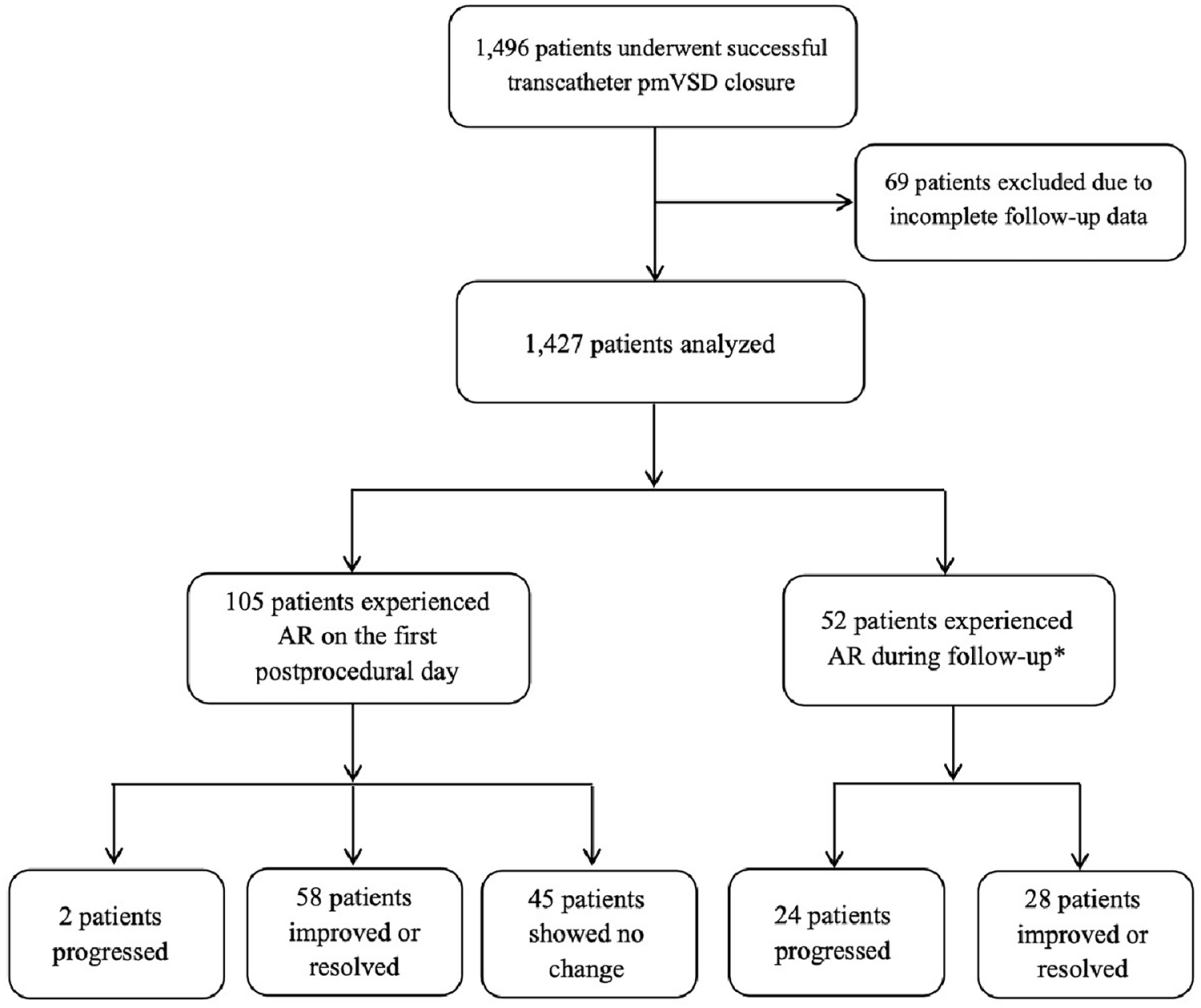
Flowchart of patient selection and outcomes. *AR occurred in 19 patients at one month, 9 at 3 months, 3 at 6 months, 7 at 1 year, and 14 more than one year post-procedure. pmVSD, perimembranous ventricular septal defect; AR, aortic regurgitation

After excluding 69 patients with incomplete follow-up data—12 who missed all postprocedural visits and 57 who, despite exceeding one year postprocedure, did not complete a full year of follow-up—a total of 1,427 patients aged 1 year and 8 months to 18 years were included (Fig 1). All patients had complete baseline and follow-up records, with a median follow-up duration of 80 months (ranging: 12—248 months). Table 1 presents the baseline characteristics and VSD-related measurements of the patients. The occurrence and clinical outcomes of major adverse events are detailed in Supplementary Table S5.

**Table 1:** Baseline patient characteristics and procedural data

	n = 1427
Female gender	718 (50.3)
Age (years)	4.43 ± 2.55
Weight (kg)	18.52 ± 8.64
Inlet diameter of VSD (mm)	6.58 ± 3.20
Outlet diameter of VSD (mm)	3.22 ± 1.28
Subaortic rim size (mm)	2.11 ± 1.45
Membranous aneurysms	880 (61.7)
Qp/Qs	2.4 ± 1.2
Device diameter (mm)	6.48 ± 2.04
Device type	
Symmetric	941 (65.9)
ADO II	201 (14.1)
Thin-waist	104 (7.3)
Eccentric	181 (12.7)
The left disk placed within the aneurysmal tissue	223 (15.6)

Data are presented as mean ± SD or frequencies (%).

VSD, ventricular septal defect; Qp/Qs, pulmonary-to-systemic flow ratio; ADO II, Amplatzer Duct Occluder II.

### Postprocedural AR

Postprocedural AR was observed in 157 cases (Table 2), including 151 new-onset and 6 aggravated cases, with 2 patients having pre-existing aortic valve prolapse (AVP). AR was noted in 105 cases on the first postprocedural day and in 52 cases during follow-up (Fig 1).

**Table 2:** Aortic regurgitation after ventricular septal defect closure

AR	Postprocedural AR	Postprocedural AR at last follow-up	Preprocedural AR	Preprocedural AR at last follow-up
Trivial	125	62	32	17
Mild	29	10	5	3
Moderate	3	2	0	0
Total	157	74	37	20

AR, aortic regurgitation.

The first patient with moderate AR was a 4-year-old girl with a 3 mm VSD and no subaortic rim. A 5-mm symmetric occluder was first trialed with temporary left-disk opening above the aortic valve; due to interference with valve motion, it was exchanged for a 6-mm eccentric occluder. On postprocedural day 1, TTE showed mild eccentric AR, which worsened by day 3. She was referred for surgery on day 4, where damage was found at the junction of the left and right coronary cusps, requiring aortic valve repair. Post-repair, only mild AR remained. The second patient underwent successful deployment of a 4-mm thin-waist occluder with the left disk opening above the aortic valve. Postprocedural TTE revealed moderate AR, with slight prolapse of the right coronary cusp into the LVOT during diastole. The third patient had a 5-mm symmetric occluder placed antegradely with the left disk opening on the LV side. Postprocedural TTE indicated enhanced echogenicity at the non-coronary cusp margin, along with moderate AR. No further intervention was needed for the latter two patients, who remain under regular follow-up.

Notably, in three additional cases, significant AR developed after occluder placement, with TTE revealing aortic valve damage, leading to abandonment of the procedure. Two of these patients had VSDs without subaortic rim and associated right coronary cusp prolapse. One case used a 12-mm eccentric occluder with the left disk opening above the aortic valve, while the other underwent two occlusion attempts. All three patients were transferred for successful surgical VSD and aortic valve repair. Intraoperative findings confirmed varying degrees of aortic valve damage: one had injury to the right coronary cusp, another to the non-coronary cusp, and the third to both.

Among the 1,427 patients, 37 had preprocedural AR (Table 2). Of these, 6 experienced worsening AR post-procedure, 18 showed improvement or resolution, and 13 had no change. Six patients had preprocedural mild right coronary cusp prolapse without AR. Of these, one developed new-onset AR, while the remaining five had no postprocedural AR. Additionally, five patients had both mild right coronary cusp prolapse and preprocedural AR. In this group, one had worsened AR, while the other four showed improvement or resolution.

### Risk factors for postprocedural AR

**Fig 2:**
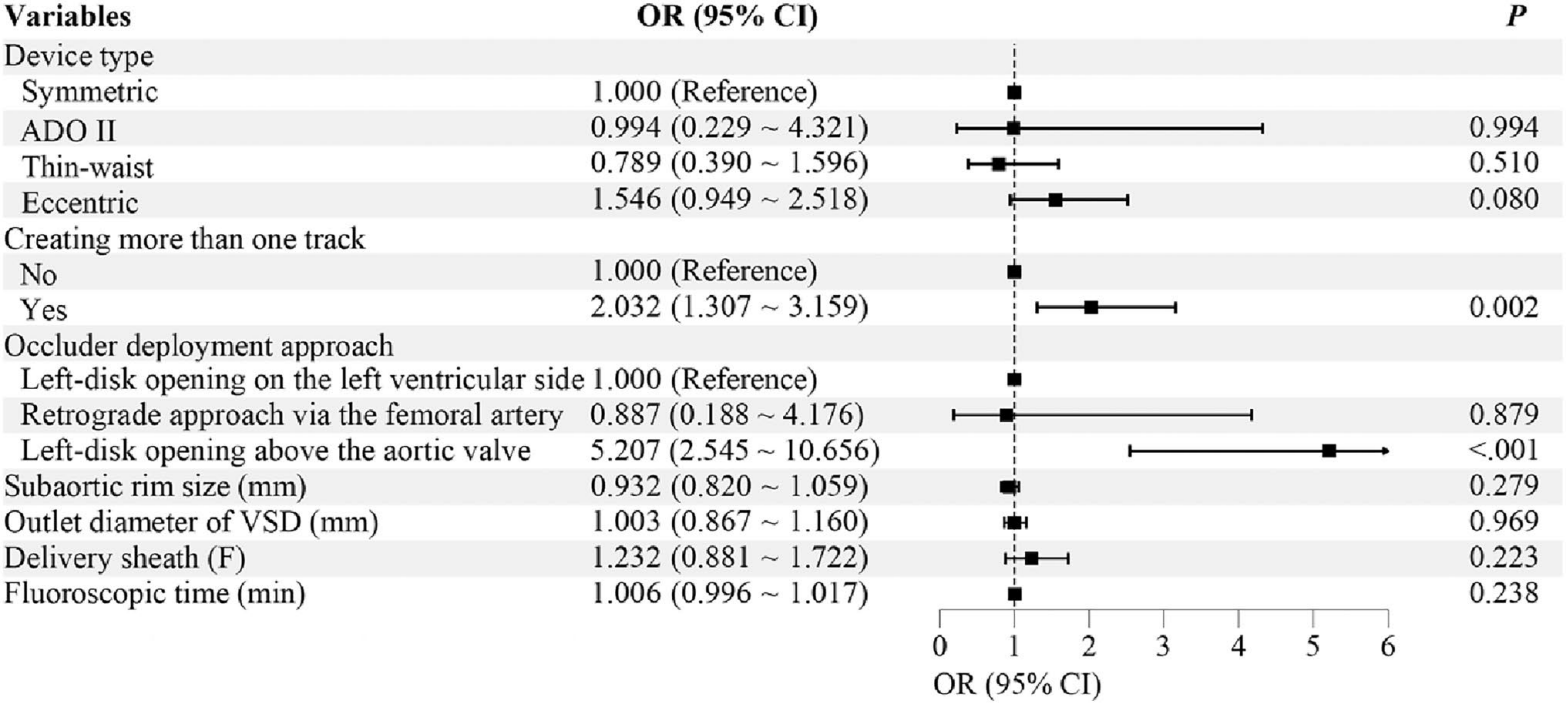
Forest plot showing independent risk factors for postprocedural aortic regurgitation after ventricular septal defect closure. OR and 95% CI were derived from multivariate logistic regression analysis. Repeated track creation and opening the left disk above the aortic valve were identified as independent risk factors for postprocedural AR. VSD, ventricular septal defect; ADO II, Amplatzer Duct Occluder II; OR, odds ratio; CI, confidence interval; AR, aortic regurgitation

Univariate analysis revealed that a smaller subaortic rim, thicker delivery sheaths, occluder type, number of tracks created, occluder deployment method, and longer fluoroscopic time (all *P* < 0.05) were associated with postprocedural AR (Table 3). Variables with *P* < 0.1 (defect outlet, subaortic rim, delivery sheath, occluder type, number of tracks created, occluder deployment method, and fluoroscopic time) were further included in multivariate analysis. After adjusting for confounders, multivariate analysis identified two independent risk factors for postprocedural AR: creating more than one track (odds ratio [OR]: 2.032; 95% confidence interval [CI]: 1.307–3.159; *P* = 0.002) and opening the left disk above the aortic valve during antegrade delivery (OR: 5.207; 95% CI: 2.545–10.656; *P* < 0.001) (Fig 2).

**Table 3:** Univariate analysis of risk factors for aortic regurgitation after ventricular septal defect closure

	AR (n=157)	None AR (n=1270)	*P* value
Female gender	86 (54.8)	632 (49.8)	0.236
Age (years)	4.49 ± 2.58	4.43 ± 2.55	0.742
Weight (kg)	18.05 ± 7.97	18.58 ± 8.72	0.467
Inlet diameter of VSD (mm)	6.56 ± 2.93	6.58 ± 3.23	0.950
Outlet diameter of VSD (mm)	3.39 ± 1.47	3.20 ± 1.26	0.067
Subaortic rim size (mm)	1.81 ± 1.50	2.14 ± 1.44	0.006
Membranous aneurysms	88 (56.1)	792 (62.4)	0.125
The left disk placed within the aneurysmal tissue	17 (10.8)	206 (16.2)	0.130
Delivery sheath (F)	6.62 ± 0.78	6.43 ± 0.83	0.007
Device type			0.001
Symmetric	97 (61.8)	844 (66.5)	
ADO II	14 (8.9)	187 (14.7)	
Thin-waist	11 (7.0)	93 (7.3)	
Eccentric	35 (22.3)	146 (11.5)	
Device diameter (mm)	6.71 ± 2.10	6.46 ± 2.03	0.137
Creating more than one track	44 (28.0)	161 (12.7)	< 0.001
Occluder deployment approach			< 0.001
Left-disk opening on the left ventricular side	129 (82.2)	1061 (83.5)	
Retrograde approach via the femoral artery	10 (6.4)	177 (13.9)	
Left-disk opening above the aortic valve	18 (11.5)	32 (2.5)	
Fluoroscopic time (min)	19.06 ± 16.67	14.59 ± 14.17	< 0.001

Data are presented as mean ± SD or frequencies (%).

AR, aortic regurgitation; VSD, ventricular septal defect; ADO II, Amplatzer Duct Occluder II.

In the early era, the results were consistent with those of the overall cohort. In the late era, a smaller subaortic rim was independently associated with postprocedural AR (OR = 0.804, 95% CI: 0.655–0.987, *P* = 0.037). The left disk opening above the aortic valve also remained a significant predictor of AR, consistent with the overall results (Supplementary Table S1-S4).

### Follow-up outcomes

During follow-up, 54.8% of patients with postprocedural AR showed improvement or resolution (Fig 1 and Table 2). Two cases of moderate AR during follow-up: In the first patient, TTE at the 3-year follow-up demonstrated mild LV enlargement, slight thickening and increased echogenicity of the right and non-coronary cusps, mild right coronary cusp prolapse during diastole, and moderate AR. In the second patient, TTE at the 5-year follow-up revealed increased echogenicity at the non-coronary cusp margin, moderate AR, and mild LV enlargement. Both patients maintained normal cardiac function and remain under close follow-up.

## Discussion

With the largest sample size to date, this study is the first to specifically explore the risk factors for AR following transcatheter pmVSD closure in children, as well as its long-term follow-up outcomes. Repeated track creation and opening the left disk above the aortic valve during antegrade delivery were identified as the primary risk factors for postprocedural AR.

AR is primarily caused by abnormalities in the aortic valve leaflets or aortic root, resulting in leaflet distortion and impaired valve closure. Severe AR can significantly affect hemodynamics by increasing the volume load on the LV. In acute AR, LV typically does not enlarge, but the sudden overload can lead to severe pulmonary congestion and reduced cardiac output. In contrast, chronic AR leads to gradual LV dilation and, over time, may cause irreversible damage. [14, 15]

Some pmVSDs located near the aortic valve can cause downward displacement of the valve leaflets during diastole due to insufficient muscular support. During systole, a rapid left-to-right shunt creates a suction effect, pulling the valve downward and causing deformation or prolapse, which may eventually lead to AR. [16] Several studies suggest that for pmVSDs with mild AVP or mild AR, transcatheter closure may be considered, as most patients show stable or improved valve function post-procedure, with only a small proportion experiencing worsening [3, 9, 10]. This is likely because the left disk of the device, positioned near the aortic root, can support the prolapsed cusp—creating a mild indentation without significantly impairing leaflet coaptation. Since effective valve closure depends on full coaptation of all three leaflets, this minimal effect usually does not impair valve function. Ideally, gentle tissue support beneath the prolapsed valve prevents wear or AR, thus preserving valve function. In this study, 83.8% of patients with preprocedural AR showed no worsening after closure, and 46.6% experienced improvement or resolution of AR. Among those with mild right coronary cusp prolapse, 81.8% had no new or worsening AR post-procedure.

To assess the severity of AR, a multiparametric approach that includes qualitative, semi-quantitative and quantitative parameters is frequently used. In this study, however, AR evaluation relied primarily on qualitative and semi-quantitative criteria, due to the retrospective design and the lack of consistent availability of quantitative indices such as vena contracta or regurgitant volume across the more than 20-year study period, particularly in its early years. While these methods are widely applied in pediatric practice and supported by current recommendations, they are subject to inter-observer variability and may limit the precision of grading, thereby influencing the reported severity and incidence of AR. Future prospective studies incorporating standardized quantitative echocardiographic measures and advanced imaging modalities will be essential to achieve a more objective and comprehensive assessment.

The incidence of AR after pmVSD closure ranges from 3.3% to 37.1% [17–20]. In this study, AR occurred in 11.0% of patients the day after the procedure, and in 5.2% at the end of follow-up.

The mechanism of AR after pmVSD closure is mainly attributed to the device impacting the aortic valve cusp or restricting the movement of the valve leaflets [21, 22]. Acute-onset AR is generally related to edema or direct injury of the aortic valve leaflets during device deployment, leading to early postprocedural valve dysfunction. In contrast, sub-acute or chronic-onset AR may be associated with valve abrasion by the device, delayed leaflet prolapse, postprocedural tissue fibrosis, or late changes in the leaflet margins and supporting structures, which gradually alter leaflets morphology and elasticity and lead to late regurgitation. Clinically, the most widely used device is the symmetrical occluder. If the subaortic rim of the pmVSD is less than 2 mm without membranous aneurysms, the left disk may interfere with the aortic valve. Theoretically, using an eccentric occluder in such situations could reduce the risk of AR. However, studies have shown that eccentric occluders are significantly associated with a higher risk of postprocedural arrhythmias. Therefore, even when the aortic rim is less than 2 mm, a symmetrical occluder is still usually preferred for initial attempts at closure. If the upper edge of the occluder is merely in contact with the aortic root or cusp but does not impair valve motion or cause regurgitation, the occluder is typically released. Nevertheless, whether AR may develop over time remains uncertain and requires long-term, regular follow-up for further verification. As previously mentioned, when the defect is in close proximity to the aortic valve, it may result in AVP. Thus, even with the use of an eccentric occluder, there remains a potential long-term risk of impaired valve closure or wear on the aortic valve. For cases with eccentric occluders, special attention should be paid to the aortic valve during follow-up.

A few studies have suggested that the ADO II offers certain advantages in closing VSDs located near the aortic valve, or even those without subaortic rims [23, 24]. This is attributed to its flexibility and ease of expansion, allowing it to move freely with the aortic valve cusps without interfering with their motion. However, the long-term impact of its use on aortic valve function remains unclear. In clinical practice, we have also attempted the use of the ADO II occluder in a small number of such cases, but no significant unique advantage in reducing AR has been observed. This may need to be further validated through studies with larger sample sizes.

This study shows that repeated attempts to establish a track during the procedure significantly increase the risk of AR. Such attempts are typically required in two situations: when a membranous aneurysm with multiple outlets causes significant residual shunting after closure, necessitating device replacement, or when a small subaortic rim leads to device interference with aortic valve function, requiring device exchange. This means repeatedly passing the catheter and delivery sheath through the aortic valve. More importantly, the delivery sheath must be advanced several times from the ascending aorta into LV. This is particularly challenging when the defect is large or the subaortic rim is small, as navigating the sheath into LV can be technically difficult and often requires repeated attempts. These factors increase the risk of aortic valve edema or even damage.

Another independent risk factor is opening the left disk above the aortic valve during antegrade delivery. Normally, except for the ADO II, the other three types of occluders are deployed via antegrade femoral vein approach within LV. However, when repeated attempts to advance the delivery sheath from the ascending aorta into LV fail, opening the left disk above the aortic valve becomes the only option. This approach involves opening the left disk above the aortic valve and then retracting it across the valve into LV. This process can cause aortic valve edema or even damage, particularly in younger, lighter patients or those with a larger left disk. Although experienced operators usually deploy the left disk in a spherical shape within the ascending aorta, the risk of aortic valve injury cannot be fully eliminated. Therefore, this approach should be avoided when possible. If necessary, the procedure should be performed by an experienced operator at a large cardiac center, with the left disk initially deployed in a spherical shape above the aortic valve to minimize potential damage.

Subgroup analysis showed that the results in the early era were consistent with those of the overall cohort, whereas in the late era, a smaller subaortic rim emerged as a new risk factor. This change may be attributed to improvements in device design and, in particular, the accumulation of operator experience, which have gradually reduced the technical risks associated with repeated track establishment. In addition, with the gradual expansion of procedural indications, a shorter defect–aortic valve distance is no longer regarded as a contraindication. Indeed, the subaortic rim in the late era was significantly smaller than that in the early era (2.28 ± 1.58 mm vs. 1.92 ± 1.27 mm, *P* < 0.001). These findings highlight the advancement of procedural techniques and the evolution of patient selection criteria, underscoring the need to continuously refine interventional strategies for VSD closure in line with technological progress and clinical practice.

In the univariate analysis, apart from a small subaortic rim, other potential risk factors for AR include the use of a larger delivery sheath and longer fluoroscopic time. For low-weight infants, a thinner delivery sheath is recommended. Fluoroscopic time is influenced by the operator’s experience, the anatomical characteristics of the defect, and the compatibility of the selected occluder. Intraoperative procedures that lead to edema of the aortic valve leaflets can also contribute to postprocedural AR. If only edema is present without structural damage, regurgitation is usually mild and often improves or resolves as the edema subsides. These factors highlight the importance of operators refining their skills, performing procedures gently and precisely, and avoiding excessive handling or rough techniques that could cause aortic valve edema or injury.

As previously noted, patients with pmVSD, accompanied by mild AVP or mild AR before procedure, appear to have a lower risk of developing AR after transcatheter closure, with only a few experiencing worsening of AR. However, these patients still face a relatively higher risk of AR or aortic valve damage due to the procedure. In this study, among the cases of aortic valve injury, two had coexisting AVP, three had the occluder deployed above the aortic valve with a larger left disk diameter, and three had defects near the right coronary leaflet with no subaortic rim.

During the procedure, the choice of occluder should be based on the the VSD’s morphology and its relationship with the aortic valve. Although left ventricular angiography may show that some defects’ edges are very close to the aortic valve, the upper margin of the defect may not be in the same plane as the right coronary or non-coronary leaflets, meaning that valve closure is typically unaffected after occluder placement. Once the occluder is positioned, TTE, left ventricular and ascending aorta angiography should confirm proper placement and normal aortic valve function before releasing the device. If the jet is minimal and central, and the occluder does not impact valve motion, the device can usually be safely released. However, if the jet is mild even greater, or eccentric, a careful assessment is required to determine whether the occluder has interfered with valve function or caused damage. In such cases, releasing the occluder is generally not recommended. A thorough echocardiographic evaluation after occluder deployment is critical to assess aortic valve anatomy and function.

During the procedure, if the occluder compresses the aortic valve or becomes lodged in the valve orifice, preventing proper valve closure and causing AR, urgent surgical device removal is necessary. This issue is typically detected promptly via echocardiographic monitoring. Additionally, severe AR resulting from aortic valve rupture due to improper procedures usually requires surgery. In this study, most cases of postprocedural AR were mild or trivial, and nearly half of the patients experienced reduced or complete resolution of regurgitation during follow-up. For those with mild or trivial AR, symptoms are generally absent, and these patients typically do not experience increased left ventricular volume load or impaired function. Some studies suggest that placing the left disk near the right coronary valve root may lead to repeated friction with the disk’s sharp edge, potentially causing valve perforation and severe AR over time. [11] Yang J. et al. reported a case of a 13-year-old girl who developed mild AR after VSD closure, which progressively worsened and ultimately required aortic valve replacement at the 2.5-year follow-up [1]. In our study, a few patients developed new or worsening AR during follow-up. Although no cases of mild or trivial AR progressed to moderate AR, it remains uncertain whether this could occur with further follow-up. Notably, among two patients with moderate AR currently under follow-up, neither has undergone surgery. However, TTE suggests potential minor aortic valve injury, with mild left ventricular enlargement observed at the 3-year and 5-year follow-ups, respectively, indicating that further surgical intervention may be necessary. Only a few patients with moderate AR required surgery, mainly those with symptoms, significant left ventricular enlargement, or impaired left ventricular systolic function. Therefore, long-term echocardiographic monitoring is essential for patients with postprocedural AR to closely track changes in regurgitation severity, cardiac chamber size, and heart function.

In the past two years, novel fully bioresorbable occluders have been introduced for clinical pmVSD closure. [25] Especially for those near or lacking a residual rim to the aortic valve, early attempts have shown promising results. Since these occluders degrade completely in approximately one year, they may reduce long-term impact on the aortic valve and potentially improve long-term patient prognosis.

## Limitations

This study employed a single-center, retrospective design. While a relatively large patient cohort was included, potential biases could not be fully controlled. Although all patients had a follow-up duration of over one year, with a median follow-up of 80 months, the varying follow-up periods may affect the stability of the results and the analysis of long-term prognosis, particularly in cases of progressively worsening postprocedural AR. Additionally, AR assessment in this study relied mainly on qualitative and semi-quantitative echocardiographic methods, without uniform quantitative parameters. These approaches are subject to inter-observer variability, which may have affected the reported severity and incidence of AR.

## Conclusion

AR is a major complication following pmVSD closure in children. Most cases of AR are mild or trivial, with a favorable long-term prognosis. However, moderate or progressive AR requires careful monitoring and timely intervention. Minimizing repeated track creations and avoiding opening the left disk above the aortic valve may help reduce the risk of AR. Additionally, a smaller subaortic rim may increase the likelihood of AR. These findings underscore the importance of procedural technique, preprocedural assessment, and regular long-term follow-up to detect AR progression early.

## Supplementary Information

Below is the link to the electronic supplementary material.

Supplementary file1 (DOCX 41 KB)
