# Supplementary material for: Aortic regurgitation following transcatheter closure of perimembranous ventricular septal defect in children: risk factors and long-term outcomes
Source: Cardiovasc Interv Ther. 2025 Nov 26;41(2):446–55. doi: 10.1007/s12928-025-01221-7 (PMC13002683; doi:10.1007/s12928-025-01221-7)
Supplement: Supplementary file 1 — Supplementary file1 (DOCX 41 KB) [file 12928_2025_1221_MOESM1_ESM.docx]

**Supplementary Table S1. Univariate analysis of risk factors for** **aortic regurgitation after ventricular septal defect closure (2002-2016)**

|  | **AR**  **（n=71）** | **None AR**  **（n=530）** | ***P* value** |
| --- | --- | --- | --- |
| Female gender | 36 (50.7) | 255 (48.1) | 0.682 |
| Age (years) | 4.93 ± 2.83 | 5.09 ± 2.90 | 0.664 |
| Weight (kg) | 19.57 ± 9.32 | 20.32 ± 9.52 | 0.533 |
| Inlet diameter of VSD (mm) | 6.43 ± 3.09 | 6.38 ± 3.44 | 0.907 |
| Outlet diameter of VSD (mm) | 3.47 ± 1.38 | 3.16 ± 1.22 | 0.050 |
| Subaortic rim size (mm) | 2.18 ± 1.70 | 2.37 ± 1.60 | 0.362 |
| Membranous aneurysms | 41 (57.7) | 337 (63.6) | 0.339 |
| The left disk placed within the aneurysmal tissue | 9 (12.7) | 106 (20.2) | 0.307 |
| Delivery sheath (F) | 6.72 ± 0.74 | 6.49 ± 0.75 | 0.017 |
| Device type |  |  | 0.031 |
| Symmetric | 39 (54.9) | 345 (65.1) |  |
| ADO II | 3 (4.2) | 36 (6.8) |  |
| Thin-waist | 7 (9.9) | 61 (11.5) |  |
| Eccentric | 22 (31.0) | 88 (16.6) |  |
| Device diameter (mm) | 6.58 ± 2.31 | 6.17 ± 2.05 | 0.126 |
| Creating more than one track | 25 (35.2) | 73 (13.8) | < 0.001 |
| Occluder deployment approach |  |  | < 0.001 |
| Left-disk opening on the left ventricular side | 59 (83.1) | 483 (91.1) |  |
| Retrograde approach via the femoral artery | 3 (4.2) | 35 (6.6) |  |
| Left-disk opening above the aortic valve | 9 (12.7) | 12 (2.3) |  |
| Fluoroscopic time (min) | 25.76 ± 20.22 | 20.31 ± 17.40 | 0.015 |

Data are presented as mean ± SD or frequencies (%).

AR, aortic regurgitation; VSD, ventricular septal defect; ADO II, Amplatzer Duct Occluder II.

**Supplementary Table S2. Multivariate logistic regression analysis of risk factors for aortic regurgitation after ventricular septal defect closure (2002-2016)**

| **Variable** | **OR** | **95%** **CI** | ***P* value** |
| --- | --- | --- | --- |
| Outlet diameter of VSD (mm) | 1.026 | 0.824-1.277 | 0.822 |
| Delivery sheath (F) | 1.349 | 0.844-2.157 | 0.211 |
| Device type |  |  |  |
| Symmetric | Ref. | Ref. | Ref. |
| ADO II | - | - | - |
| Thin-waist | 0.845 | 0.340-2.100 | 0.717 |
| Eccentric | 1.703 | 0.902-3.216 | 0.100 |
| Creating more than one track | 2.954 | 1.567-5.567 | 0.001 |
| Occluder deployment approach |  |  |  |
| Left-disk opening on the left ventricular side | Ref. | Ref. | Ref. |
| Retrograde approach via the femoral artery | - | - | - |
| Left-disk opening above the aortic valve | 9.001 | 3.368-24.050 | < 0.001 |
| Fluoroscopic time (min) | 1.001 | 0.987-1.015 | 0.900 |

VSD, ventricular septal defect; ADO II, Amplatzer Duct Occluder II; OR, odds ratio; CI, conﬁdence interval.

**Supplementary Table S3. Univariate analysis of risk factors for aortic regurgitation after ventricular septal defect closure (2017-2023)**

|  | **AR**  **（n=86）** | **None AR**  **（n=740）** | ***P* value** |
| --- | --- | --- | --- |
| Female gender | 50 (58.1) | 377 (50.9) | 0.206 |
| Age (years) | 4.14 ± 2.30 | 3.95 ± 2.15 | 0.446 |
| Weight (kg) | 16.79 ± 6.45 | 17.33 ± 7.87 | 0.538 |
| Inlet diameter of VSD (mm) | 6.67 ± 2.81 | 6.72 ± 3.06 | 0.884 |
| Outlet diameter of VSD (mm) | 3.34 ± 1.54 | 3.22 ± 1.28 | 0.453 |
| Subaortic rim size (mm) | 1.50 ± 1.25 | 1.99 ± 1.29 | 0.001 |
| Membranous aneurysms | 47 (54.7) | 455 (61.5) | 0.219 |
| The left disk placed within the aneurysmal tissue | 8 (9.3) | 100 (13.5) | 0.355 |
| Delivery sheath (F) | 6.53 ± 0.81 | 6.39 ± 0.89 | 0.135 |
| Device type |  |  | 0.073 |
| Symmetric | 58 (67.4) | 500 (67.6) |  |
| ADO II | 11 (12.8) | 150 (20.3) |  |
| Thin-waist | 4 (4.7) | 32 (4.3) |  |
| Eccentric | 13 (15.1) | 58 (7.8) |  |
| Device diameter (mm) | 6.83 ± 1.92 | 6.66 ± 2.00 | 0.464 |
| Creating more than one track | 19 (22.1) | 88 (11.9) | 0.008 |
| Occluder deployment approach |  |  | < 0.001 |
| Left-disk opening on the left ventricular side | 70 (81.4) | 578 (78.1) |  |
| Retrograde approach via the femoral artery | 7 (8.1) | 142 (19.2) |  |
| Left-disk opening above the aortic valve | 9 (10.5) | 20 (2.7) |  |
| Fluoroscopic time (min) | 13.53 ± 10.24 | 10.50 ± 9.39 | 0.005 |

Data are presented as mean ± SD or frequencies (%).

AR, aortic regurgitation; VSD, ventricular septal defect; ADO II, Amplatzer Duct Occluder II.

**Supplementary Table S4. Multivariate logistic regression analysis of risk factors for aortic regurgitation after ventricular septal defect closure (2017-2023)**

| **Variable** | **OR** | **95% CI** | ***P* value** |
| --- | --- | --- | --- |
| Subaortic rim size (mm) | 0.804 | 0.655-0.987 | 0.037 |
| Device type |  |  |  |
| Symmetric | Ref. | Ref. | Ref. |
| ADO II | 1.074 | 0.209-5.512 | 0.932 |
| Thin-waist | 0.809 | 0.264-2.486 | 0.712 |
| Eccentric | 1.147 | 0.538-2.447 | 0.722 |
| Creating more than one track | 1.433 | 0.760-2.703 | 0.266 |
| Occluder deployment approach |  |  |  |
| Left-disk opening on the left ventricular side | Ref. | Ref. | Ref. |
| Retrograde approach via the femoral artery | 0.453 | 0.073-2.791 | 0.393 |
| Left-disk opening above the aortic valve | 3.047 | 1.025-9.057 | 0.045 |
| Fluoroscopic time (min) | 1.017 | 0.995-1.039 | 0.125 |

ADO II, Amplatzer Duct Occluder II; OR, odds ratio; CI, conﬁdence interval.

**Supplementary Table S5. The occurrence and clinical outcomes of major adverse events**

| Major adverse events | Number of Patients | Proportion of Patients | Timing Relative to Procedure | **Clinical Outcome** |
| --- | --- | --- | --- | --- |
| cAVB | 5 | 0.3% | 3 early (≤1 week), 2 late (2 & 4 years) | Three patients received permanent pacemaker implantation, one reverted to type I second-degree AVB with IRBBB, one reverted to CRBBB but died of an Adams-Stokes attack secondary to cAVB at 40 days post-procedure. |
| CLBBB | 28 | 1.9% | 26 early (≤1 week), 2 late (both 6 months) | Four patients underwent surgery with occluder removal and VSD repair at 6, 6, 13, and 40 days post-procedure, all recovering normal conduction; 19 recovered with medical therapy; 5 did not recover, including 1 requiring CRT. |
| New-onset TR that required surgical repair | 2 | 0.1% | 1 day and 2 years post-procedure | Surgical tricuspid valvuloplasty was performed at 12 days and 2 years post-procedure. |
| Tricuspid stenosis requiring surgical intervention | 1 | 0.07% | Immediately after device release | Emergency cardiac surgery |
| Device embolization | 1 | 0.07% | 1 day post-procedure | Transcatheter removal |
| Death | 1 | 0.07% | 11 days post-procedure | Due to postprocedural diffuse subarachnoid hemorrhage |

AVB = atrioventricular block; cAVB = complete atrioventricular block; IRBBB = incomplete right bundle branch block; CRBBB = complete right bundle branch block; CLBBB = complete left bundle branch block; VSD = ventricular septal defect; CRT = cardiac resynchronization therapy; TR = tricuspid regurgitation

**Supplementary Table S6. Univariate analysis of risk factors for persistent or surgically treated aortic regurgitation after ventricular septal defect closure**

|  | **AR**  **（n=74）** | **None AR**  **（n=1353）** | **P value** |
| --- | --- | --- | --- |
| Female gender | 39 (52.7) | 679 (50.2) | 0.673 |
| Age (years) | 4.59 ± 2.66 | 4.42 ± 2.55 | 0.593 |
| Weight (kg) | 18.04 ± 7.56 | 18.54 ± 8.70 | 0.626 |
| Inlet diameter of VSD (mm) | 6.60 ± 3.02 | 6.57 ± 3.21 | 0.949 |
| Outlet diameter of VSD (mm) | 3.46 ± 1.62 | 3.20 ± 1.26 | 0.095 |
| Subaortic rim size (mm) | 1.67 ± 1.46 | 2.13 ± 1.44 | 0.007 |
| Membranous aneurysms | 42 (56.8) | 838 (61.9) | 0.372 |
| The left disk placed within the aneurysmal tissue | 9 (12.2) | 214 (15.8) | 0.569 |
| Delivery sheath (F) | 6.49 ± 0.83 | 6.45 ± 0.83 | 0.702 |
| Device type |  |  | 0.476 |
| Symmetric | 44 (59.5) | 898 (66.4) |  |
| ADO II | 10 (13.5) | 190 (14.0) |  |
| Thin-waist | 7 (9.5) | 97 (7.2) |  |
| Eccentric | 13 (17.6) | 168 (12.4) |  |
| Device diameter (mm) | 6.74 ± 2.27 | 6.47 ± 2.03 | 0.264 |
| Creating more than one track | 25 (33.8) | 180 (13.3) | < 0.001 |
| Occluder deployment approach |  |  | < 0.001 |
| Left-disk opening on the left ventricular side | 53 (71.6) | 1137 (84.0) |  |
| Retrograde approach via the femoral artery | 7 (9.5) | 180 (13.3) |  |
| Left-disk opening above the aortic valve | 14 (18.9) | 36 (2.7) |  |
| Fluoroscopic time (min) | 19.34 ± 16.51 | 14.85 ± 14.38 | 0.010 |

Data are presented as mean ± SD or frequencies (%).

AR, aortic regurgitation; VSD, ventricular septal defect; ADO II, Amplatzer Duct Occluder II.

**Supplementary Table S7. Multivariate logistic regression analysis of risk factors for persistent or surgically treated aortic regurgitation after ventricular septal defect closure**

| **Variable** | **OR** | **95% CI** | **P value** |
| --- | --- | --- | --- |
| Outlet diameter of VSD (mm) | 1.120 | 0.938-1.339 | 0.211 |
| Subaortic rim size (mm) | 1.004 | 0.990-1.018 | 0.058 |
| Creating more than one track | 2.698 | 1.489-4.890 | 0.001 |
| Occluder deployment approach |  |  |  |
| Left-disk opening on the left ventricular side | Ref. | Ref. | Ref. |
| Retrograde approach via the femoral artery | 1.340 | 0.566-3.174 | 0.506 |
| Left-disk opening above the aortic valve | 8.619 | 4.208-17.651 | < 0.001 |
| Fluoroscopic time (min) | 1.004 | 0.990-1.018 | 0.580 |

VSD, ventricular septal defect; OR, odds ratio; CI, conﬁdence interval.
